# Aging triggers mitochondrial, endoplasmic reticulum, and metabolic stress responses in the heart

**DOI:** 10.20517/jca.2024.17

**Published:** 2025-02-18

**Authors:** Sakthijothi Muthu, Zinnia Tran, Jayapalraja Thilagavathi, Tanvi Bolarum, Edouard I. Azzam, Carolyn K. Suzuki, Venkatesh Sundararajan

**Affiliations:** 1 Department of Physiology, Pharmacology and Toxicology, School of Medicine, West Virginia University, Morgantown, WV 26506, USA.; 2 Department of Microbiology, Biochemistry and Molecular Genetics, Rutgers-New Jersey Medical School, Newark, NJ 07103, USA.; 3 Department of Radiology, Rutgers New Jersey Medical School, Cancer Center, Newark, NJ 07101, USA.

**Keywords:** Aging, heart, mitochondrial stress, endoplasmic reticulum stress, oxidative stress

## Abstract

**Introduction:**

Aging is a multifaceted biological process characterized by a progressive decline in cellular and tissue function. It significantly impacts the cardiovascular system and contributes to the onset of cardiovascular diseases. The mitochondria (mt) and the endoplasmic reticulum (ER) play synergistic roles in maintaining cellular homeostasis and energy production in the heart. Nevertheless, their response to cardiac aging is not well known.

**Aim:**

This study explores mt and ER stress responses and their associated factors, such as metabolic, cellular, and autophagic stress, in cardiac aging.

**Methods and Results:**

We utilized 10- and 25-month-old CBA/CaJ mice to evaluate mt, ER, and their associated factors, such as metabolic, cellular, and autophagic stress responses. We studied the gene expression for mitochondrial biogenesis, mt and ER stress response, autophagy and metabolic markers, and activating transcription factors that mediate cellular stress responses. We found no significant difference in mtDNA content and the mRNA expression of the mt transcription factor, *Tfam*; however, selective mtDNA genes, such as *mt-Cytb* and mt-Co2, showed significant induction in 25-month-aged compared to 10-month-young hearts. Interestingly, genes of several mitochondrial stress response proteases and their components, including *Lonp1, Yme1l1, Afg3l2,* and *Spg7*, were significantly induced, with a substantial induction of *Clpp* and *Clpx*. However, age-associated differences were not observed in the induction of mt chaperones (*Hspa9* and *Hspd1*), but significant induction of *Dnaja2*, a mitochondrial co-chaperone, was observed. The ER stress transcription factors *Xbp1* and *Atf6* were markedly induced in aged hearts, accompanied by decreased expression of ER stress chaperone *Hsp90b* with no change in *Hspa5* and *Dnajb9* chaperones. However, induction of *Dnm1l* was significant, whereas *Mfn1* and *Fis1* were downregulated in contrast to *Mfn2*, suggesting dysregulated mitochondrial dynamics in the aged heart with no change in autophagy and metabolic stress regulators observed. Furthermore, aged hearts showed significantly increased oxidative damage as evidenced by elevated lipid peroxidation (4-HNE) levels.

**Conclusion:**

These findings demonstrate that aging triggers mt, ER, and oxidative stress in the heart, which over time leads to the accumulation of oxidative damage, causing cellular impairment, highlighting these pathways as potential therapeutic targets for mitigating age-related cardiac dysfunction.

## Graphical Abstract

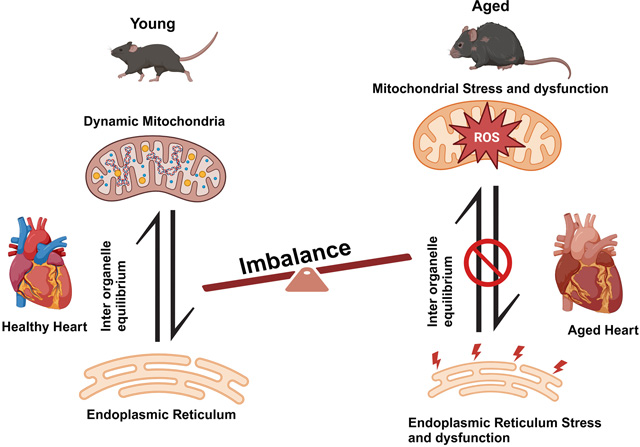

## INTRODUCTION

Aging is a multifaceted biological process of progressive decline in cellular and tissue function, significantly affecting the cardiovascular system. Advancing age is a critical contributor to the onset of cardiovascular diseases, which rank among the leading causes of morbidity and mortality worldwide. Cardiac aging is characterized by numerous changes at the cellular level, including alterations in mitochondrial (mt) and endoplasmic reticulum (ER) functions. These changes are particularly critical in cardiac health, as the heart is an organ with high metabolic demands and relies on adequate cellular homeostasis and energy production^[1]^.

Mitochondria play a central role in energy production, especially in high-energy-demanding tissues like the heart. They are responsible for most ATP generation through oxidative phosphorylation, which is a crucial process for maintaining cardiac function. Mitochondrial dysfunction, characterized by reduced efficiency in ATP production and increased production of reactive oxygen species, has been implicated in cardiac aging and the pathogenesis of age-related cardiovascular diseases^[2,3]^. The ER is another vital organelle responsible for protein folding, maturation, and trafficking. ER stress, resulting from the accumulation of misfolded proteins, triggers a protective response known as the unfolded protein response (UPR^ER^). The UPR^ER^ aims to restore ER function but can induce apoptosis if the stress is severe or prolonged^[4]^. Previous research has focused on general dysfunction in these organelles with age^[5,6]^. Despite the acknowledged importance of mt and ER in cardiac health, their interaction and contribution to cardiac aging need to be better understood. This knowledge gap has impeded the development of targeted interventions to mitigate the effects of aging on the heart.

This study aims to determine whether mt and ER stresses, along with their associated metabolic, cellular, and autophagic stress responses, play a role in cardiac aging by studying critical genetic components of stress associated with them. Using CBA/CaJ mice as a model, we assessed changes in mitochondrial biogenesis, mt and ER stress responses, autophagy and metabolic alterations, and activation of transcription factors (ATFs) in response to aging by comparing 10-month-old (representative of young adulthood) and 25-month-old (representative of advanced age) mice; thus, we provide insights into the specific cellular changes that occur in the heart as a result of aging. Here, we focused on investigating gene induction by measuring transcripts rather than proteins, as this enables the identification of early and dynamic molecular changes driving aging-related mitochondrial and ER stress while providing a comprehensive understanding of regulatory pathways, including transcriptional activators and stress responses. This approach is more sensitive, scalable, and targeted toward upstream mechanisms, offering valuable insights for therapeutic interventions. In this study, our findings contribute to the roles of these organelles, with potential implications for developing therapeutic strategies mitigating aging-associated cardiac dysfunction.

## MATERIAL AND METHODS

### Animals

**Figure 1.**
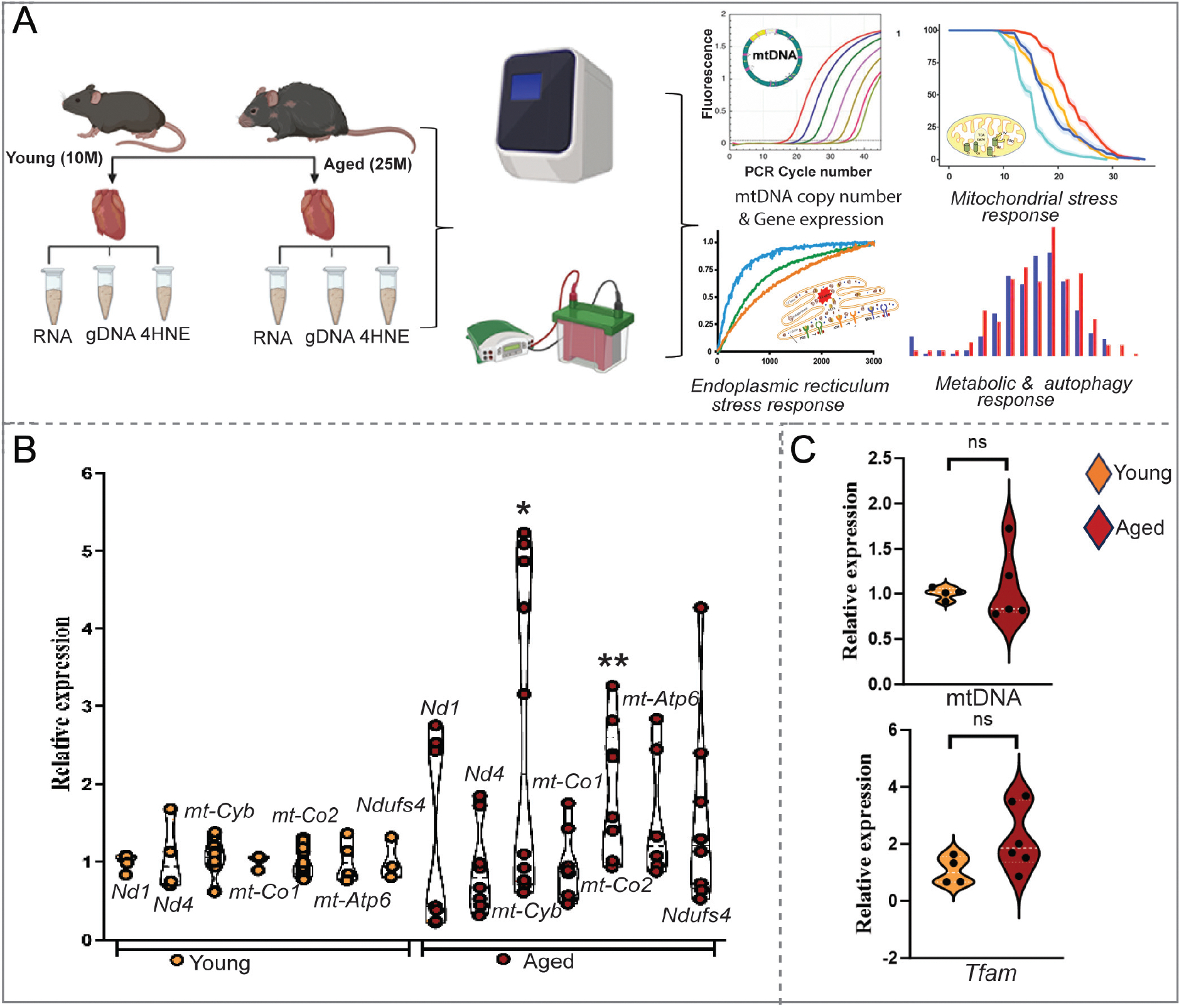
Cardiac aging alters mitochondrial biogenesis. (A) Schematic representation of the study comparing young (10-month) *vs*. old (25-month) CBA/CaJ mice hearts subjected to genomic DNA, mRNA, and protein isolation and investigation of mt biogenesis, mt and ER stress response, autophagy, and metabolic alterations. This schematic figure was created using BioRender (www.app.biorender.com). (B) Relative mRNA expression levels of mtDNA-encoded (*Nd1, Nd4, mt-Cytb, mt-Co2, mt-Atp6*) and nDNA-encoded mitochondrial *Ndufs4*. (C) Relative mtDNA content (top) normalized to *Tert*, and mRNA expression levels of *Tfam* normalized to *Gapdh* in young *vs*. old hearts. Experiments were conducted in triplicate, and relative expression levels were plotted. Values are presented as mean ± SEM (*n* = 3–10). *P < 0.05, **P < 0.01, by an unpaired Student’s *t*-test, using GraphPad Prism software.

We employed 10- and 25-month-old CBA/CaJ male mice in this study. All mice were procured from Jackson Laboratories as part of a National Aeronautics and Space Administration (NASA) study. The control animals in the study were divided into two experiments based on age. Animals were fed a standard laboratory rodent chow and drinking water ad libitum, with a 12-h light/dark cycle under a temperature-controlled environment. Quarantine procedures and animal maintenance were followed according to the recommendations of the regulatory policies of the institutional animal care and use committee (IACUC) of Rutgers Biomedical and Health Sciences, Newark, New Jersey, USA. Animals were euthanized as per the IACUC approval procedure, and the hearts were harvested, snap-frozen, and stored at −80 °C until further analysis. Later, the stored hearts were ground with dry ice and aliquoted for respective assays. The graphical Figure 1A shows the experimental design of the study.

### Determination of mitochondrial DNA content

Relative levels of mitochondrial DNA copy number (mtDNA-CN) were measured using a real-time qPCR assay. Genomic DNA was isolated from mouse heart tissue. For this, 0.5 mL of lysis buffer (100 mM Tris-HCl pH 8.5, 5 mM EDTA, 0.2% SDS, 200 mM NaCl, and 100 μg/mL proteinase K) was added to the heart tissue homogenate. The mixture was then incubated at 37 °C for several hours with constant agitation to ensure thorough digestion. Post-digestion, an equal volume of isopropanol was added to the lysate to complete DNA precipitation. The precipitated DNA was then recovered, washed twice with ice-cold 70% ethanol, air-dried, and resuspended in TE buffer for subsequent analysis. 100 ng of the isolated genomic DNA was employed to quantify relative mtDNA content by amplifying both the *mt-Cytb* gene (Mm04225271_g1, Thermo Fisher Scientific) and the nuclear *Tert* gene (reference) (Mm00653609_cn, Thermo Fisher Scientific). Amplification was performed using Applied Biosystems or Bio-Rad universal PCR master mix. The standard qPCR conditions included an initial denaturation at 95 °C for 10 min, followed by 40 cycles of denaturation at 95 °C for 15 s and annealing/extension at 60 °C for 1 min. All reactions were carried out in technical triplicate. The relative fold quantitation of the mtDNA copy number was calculated using the ΔΔCt method. All raw values are provided in Supplementary Table 1.

### Gene expression assays by Q-PCR

Total RNA was extracted from the heart homogenate using the Qiagen RNeasy mini kit for fibrous tissue (Cat# 74704, Qiagen Inc.). The concentration of RNA (ng/μL) in each sample was measured using a Nanodrop Spectrophotometer (Nanodrop Technologies Inc., USA). cDNA was synthesized from RNA (100–500 ng) using a high-capacity cDNA reverse transcription kit (Cat #4368814, Applied Biosystems) followed by qRT-PCR. We employed TaqMan-based gene expression assays for each gene target, as listed in Table 1. The relative fold expression levels were normalized with 18S and quantified using the ΔΔCt method. All raw values are provided in Supplementary Table 1.

### Western blot

We employed western blot to identify and quantify 4-HNE levels in the protein extracts from the hearts. One aliquot of the ground tissue was washed with cold phosphate-buffered saline (PBS) on the analysis day and quickly spun at 4 °C to remove blood. The protein lysis buffer with Halt protease and phosphatase single-use inhibitor cocktail at 2X final concentration (Thermo Scientific) was added to the homogenate and placed on ice for 20 min, followed by centrifugation at 14,000 rpm or 15 min at 4 °C. The supernatant was collected, and the total protein concentration was determined using the Bradford protein estimation assay (Bio-Rad, Cat# 23200) reagent. Extracted proteins (30 μg) were electrophoretically separated using 12% sodium dodecyl sulfate-polyacrylamide gels and then transferred to nitrocellulose membrane. Confirmation of protein transfer was ensured by Ponceau staining, followed by the membrane being de-stained by washing with deionized water (DI) and PBS. The membrane was blocked with 3% blocking buffer (15 g of dry milk powder in 500 mL of 1X PBS) for 60 min, followed by incubating at 4 °C overnight with anti-4HNE (ab46545, Abcam) primary antibody diluted at 1:1,000 μL in 1X TBST (Tris-Buffered Saline with Tween 20). The next day, the membrane was washed with PBS for 1 h, followed by incubation with an anti-rabbit secondary antibody conjugated to horseradish peroxidase in a blocking buffer of 3% milk in PBS. The immunoblotted membrane was developed using enhanced chemiluminescent Western Bright ECL spray (cat# K-12049-D50, Advansta) and imaged using the Bio-Rad ChemiDoc imaging system. The intensity of 4HNE bands was quantified using Quantity One software (Bio-Rad). The units were expressed as arbitrary. The original blot is provided in Supplementary Figure 1.

### Statistical analysis

The significant differences between young and aged groups were compared using the student’s *t*-test using GraphPad Prism software, and *p* < 0.05 was considered statistically significant.

## RESULTS

### Differential expression of mitochondrial biogenesis markers in the aging heart

Mitochondrial biogenesis is critical for maintaining the bioenergetic and functional equilibrium of the aging heart^[7]^. To explore changes in mitochondrial biogenesis in the hearts of young and aged mice, we examined markers of mitochondrial biogenesis: the relative mtDNA copy number, the transcript levels of mtDNA-encoded genes (*mt-Co2, mt-Cytb, mt-Nd1, mt-Nd4, and mt-Atp6*) and nuclear-encoded gene *Ndufs4*, which is a subunit of Complex I of the electron transport chain, and mitochondrial transcription factor A (*Tfam*), which is a central regulator of mtDNA maintenance, replication, and gene expression. A robust induction of mRNA levels of the mtDNA-encoded *mt-Co2* and *mt-Cyb* was observed in aging hearts compared to their younger counterparts [Figure 1B]. Furthermore, *Ndufs4* mRNA levels showed a substantial upregulation in the aged heart compared to the younger one [Figure 1B], showing gene-dependent changes during aging. Another mtDNA-encoded gene, *mt-Atp6*, showed a substantial increase, but no significant change in *mt-Nd1* and *mt-Nd4* mRNA expression in aged hearts compared to younger hearts [Figure 1B]. Interestingly, no significant difference in the relative mtDNA content and *Tfam* gene expression was noted in the aged hearts compared to their younger counterparts [Figure 1C].

### Induction of mitochondrial stress response in the aging heart

**Figure 2.**
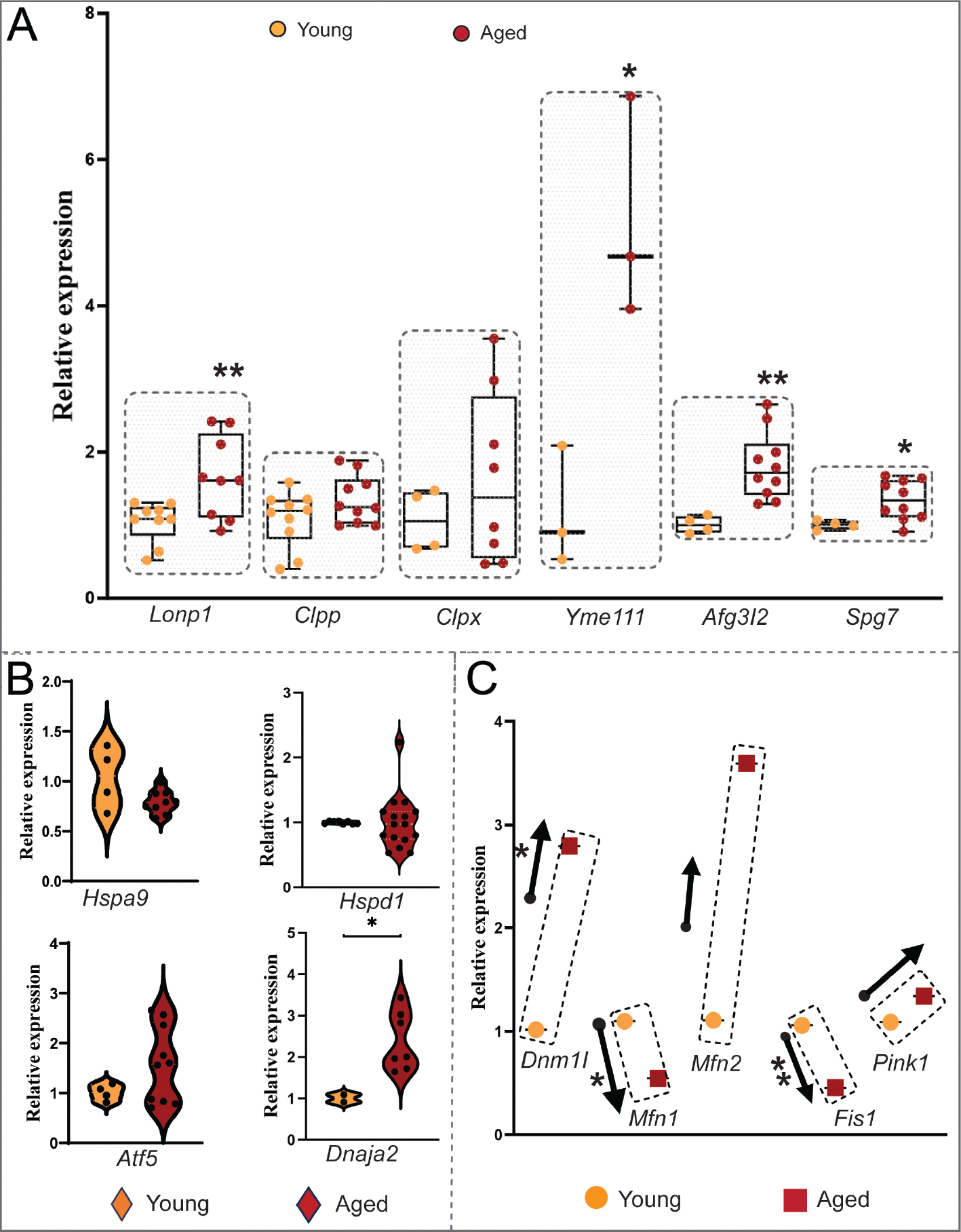
Aging induces mitochondrial stress response and changes mt dynamics in the heart. (A) Relative mRNA expression levels of mt ATP-dependent proteases and its components in aged (25-month) *vs*. young (10-month) CBA/CaJ mice hearts, normalized to *Gapdh*. (B) Relative mRNA expression levels of mt chaperones and *Atf5* in aged *vs*. young hearts normalized to *Gapdh*. (C) Relative mRNA expression levels of mt dynamic regulators in aged *vs*. young hearts normalized to *Gapdh*. Relative expression values were represented as mean ± SEM (*n* = 3–10). *P < 0.05, **P < 0.01 are considered significant, calculated by an unpaired Student’s *t*-test using GraphPad Prism software.

With age, mitochondria can undergo structural and functional changes, and contribute to cellular stress and dysfunction^[8]^. Mitochondrial protein quality control is one of the critical components in maintaining cellular homeostasis. Mitochondrial ATP-dependent proteases and chaperones are essential for mitochondrial proteostasis by promoting correct protein folding, selectively degrading misfolded, misassembled, and damaged polypeptides, and preventing protein aggregation^[9]^. We investigated the relative transcript levels of the mitochondrial stress response proteases and chaperones. We found significant upregulation in the transcript expression of mitochondrial ATP-dependent proteases *Lonp1, Yme1l1, Afg3l2,* and *Spg7* in the older *vs*. younger mouse hearts [Figure 2A]. However, *CLPXP*, another ATP-dependent protease composed of two protein complexes, *CLPX* and *CLPP*, showed substantial induction but not significant [Figure 2A]. No change in the mRNA expression level of mitochondrial chaperone, *Hspa9*, and *Hspd1* was observed, but the mt co-chaperone *Dnaja2* showed significant induction along with the substantial induction of activating transcription factor 5 (*Atf5*) in aged hearts, and these factors are associated with the mitochondrial stress response [Figure 2B].

### Dysregulation of mitochondrial dynamics in the aged heart

**Figure 3.**
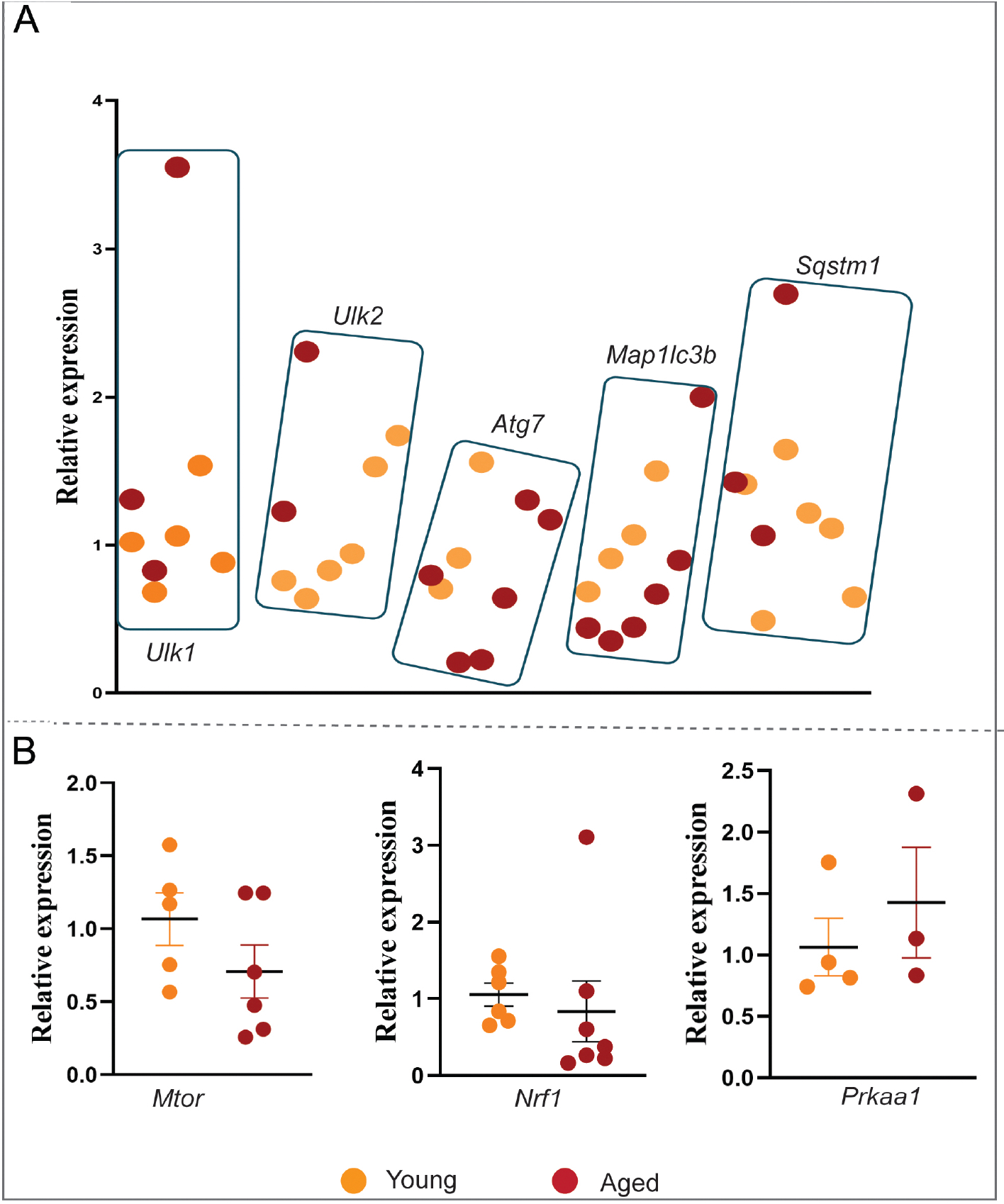
Impaired metabolic and autophagy regulation in cardiac aging. (A) Reduced mRNA expression levels of autophagy regulators in aged (25-month) *vs*. young (10-month) CBA/CaJ mice hearts, normalized to *Gapdh*. (B) Top relative mRNA expression levels of essential metabolic regulators (*Mtor, Nrf1,* and *Prkaa1*) in aged (25- month) *vs*. young (10-month) CBA/CaJ mice hearts, normalized to *Gapdh*. Relative expression values were represented as mean ± SEM (*n* = 3–10). Significant difference was calculated by an unpaired Student’s *t*-test using GraphPad Prism software.

Mitochondrial dynamics that involve the selective culling of damaged mitochondria are critical during cardiac aging^[10]^. We examined the mRNA gene expression of proteins involved in autophagy and mitophagy, which is a form of autophagy that specifically degrades damaged or stressed mitochondria: PTEN-induced putative kinase1 (PINK1), a mitochondrial serine/threonine-protein kinase involved in mitophagy, the mitochondrial fission proteins FIS1 and the DNM1L-GTPase, and the fusion proteins MFN1 and MFN2^[11]^. We found that induction of *Dnm1l* was significant, whereas *Mfn1* and *Fis1* were significantly downregulated with substantial but non-significant induction of *Mfn2* [Figure 2C]. On the other hand, *Pink1*, which plays a role in removing damaged mitochondria (mitophagy), is substantially induced in the aged heart compared to its younger counterpart [Figure 2C]. We also investigated autophagy regulation, a global mass degradation or recycling system that plays a crucial role in maintaining cellular homeostasis. We observed no substantial changes in the relative expression of autophagy initiator Unc-51-like autophagy activating kinase 1(*Ulk1), Ulk2,* and *Sqstm1* in aged mice hearts compared with young mice hearts [Figure 3A]. Likewise, the ubiquitin-like conjugation system *Atg7*, a critical regulator of autophagosome maturation, showed no change in the aged hearts compared to the younger ones [Figure 3A].

### Impaired metabolic regulators in cardiac aging

One of the key changes that could happen during aging is metabolic alterations. Therefore, we also investigated potential age-related changes in critical metabolic regulators such as *mTOR* and *Nrf1*, which are essential for protein synthesis, cellular growth, and oxidative stress response. At the same time, *Ampk1* plays a pivotal role in energy balance. Although insignificant, we observed a substantial decrease in the expression of *mTOR and Nrf1* genes in aged hearts but no change in *Prkaa1* expression [Figure 3B]. These findings suggest that the decline in *mTOR* and *Nrf1* expressions might contribute to the impaired metabolic functions associated with cardiac aging.

### Induction of selective ER stress response pathway in the aging heart

**Figure 4.**
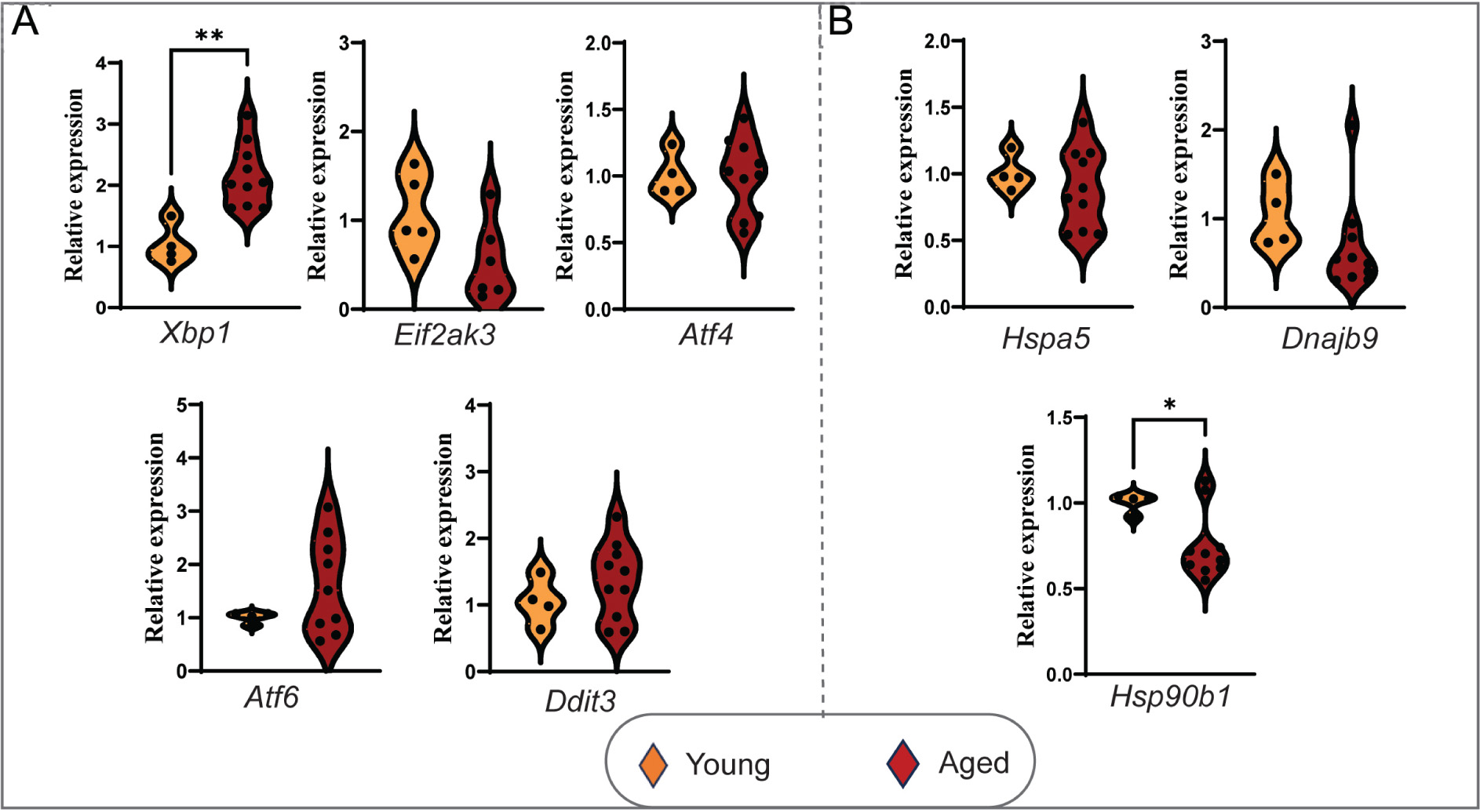
Aging induces selective endoplasmic reticulum stress response in the heart. (A) Relative mRNA expression levels of ER stress response factors in aged (25-month) *vs*. young (10-month) CBA/CaJ mice hearts, normalized to *Gapdh*. (B) Relative mRNA expression levels of ER stress response factors in aged (25-month) *vs*. young (10-month) CBA/CaJ mice hearts, normalized to *Gapdh*. Relative expression values were represented as mean ± SEM (*n* = 3–10). *P < 0.05, **P < 0.01 are considered significant, calculated by an unpaired Student’s *t*-test using GraphPad Prism software.

During cardiac aging, ER is a critical organelle involved in protein synthesis and folding, and is susceptible to dysfunction, leading to the accumulation of misfolded proteins that could trigger ER stress response^[12]^. Moreover, in many instances, mt and ER coordinate cellular functions^[13]^. In our investigation, we observed a significant induction of *Xbp1* (X-box binding protein 1), which is a transcription factor that regulates the expression of many genes involved in ER stress response, suggestive of an activated adaptive response through *Xbp1* to address the emerging ER stress milieu in the aging heart [Figure 4A]^[14,15]^. However, the induction of ER stress transcription factors *Atf4* and *Eif2ak3* (*Perk*) were not altered. Other essential stress transcription factors, such as *Chop (Ddit3)* and *Atf6,* were found to be substantially but not significantly induced [Figure 4B]. At the same time, induction of *Hsp90b1*(*Grp94*) was significantly reduced, and no change in the mRNA expression of *Hspa5* and *Dnajb9* was observed [Figure 4B].

### Increased oxidative markers in the aging heart

**Figure 5.**
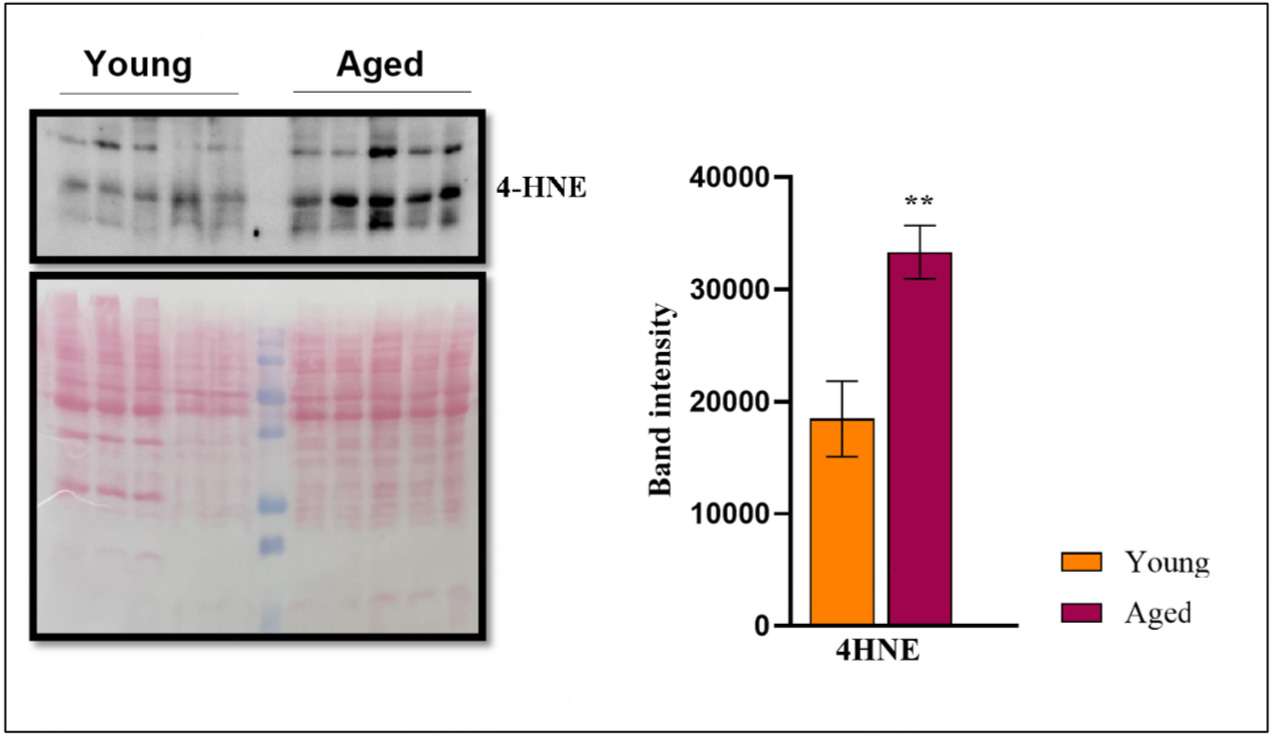
Increased oxidative stress in cardiac aging. Left: Immunoblot showing the expression of 4-HNE in aged (25-month) *vs*. young (10-month) CBA/CaJ mice hearts and corresponding Ponceau stained loading control blot. Right: Densitometry quantification of the 4-HNE levels normalized to ponceau staining. Relative expression values were represented as mean ± SEM (*n* = 5). **P < 0.01 is considered significant, calculated by an unpaired Student’s *t*-test using GraphPad Prism software.

Oxidative stress induced damage are critical phenomena associated with aging, affecting the longevity and functionality of tissues and organs^[16]^. To investigate whether oxidative damage occurs during cardiac aging, we examined lipid peroxidation, a hallmark of tissue oxidative damage. 4-hydroxy 2-nonenal (4-HNE) is generated in the oxidation of lipids containing polyunsaturated omega-6 fatty acids, and is the most abundant and stable end product of lipid peroxidation, a widely accepted stable marker for oxidative stress. We found that aged hearts exhibited significantly elevated levels of 4-HNE-modified proteins compared to younger controls [Figure 5].

## DISCUSSION

Aging is a major risk factor for cardiac dysfunction in older adults^[17]^. Mitochondria and ER are two important dynamic organelles crucial for cardiac function, and whether they are affected in aging and contribute to cardiac diseases is not well known. Our findings shed light on the potential contribution of mt and ER stress to the pathology of cardiac function during aging. The novelty of our study lies in the comprehensive elucidation of the interplay between mitochondrial and ER stress responses during cardiac aging, specifically highlighting the upregulation of mitochondrial ATP-dependent proteases such as Lonp1 and the ER stress mediator Xbp1. These findings provide new insights into the adaptive stress mechanisms in aged hearts and identify potential therapeutic targets to mitigate aging-associated cardiac dysfunction, bridging a critical gap in understanding the molecular basis of cardiac aging.

Mitochondrial biogenesis is the coordinated synthesis of nuclear and mitochondrial DNA-encoded mitochondrial protein synthesis, and mitochondrial transcription factor A (TFAM) regulates the transcription and replication rate of mitochondrial DNA with other factors, and also controls its availability to relevant proteins^[18,19]^. Our findings comparing young and aged hearts show no difference in mtDNA content but an increase in the expression of selective mtDNA-encoded genes such as *mt-Co2, mt-Cytb,* and *mt-Atp6*. This differential gene expression pattern is consistent with the previous study, indicating that aging may lead to selective upregulation and downregulation of mitochondrial-encoded genes as an adaptive response^[20]^. Although the total mtDNA content remains unchanged, the increased expression of slect mtDNA-encoded genes suggests that aged hearts optimize the function of exisitng mitochondria rather than increasing mtDNA content^[21]^.

As mitochondrial biogenesis is essential to adapt to the demand during aging, it is also imperative to maintain the protein quality within mitochondria, which is evident from our findings that a group of highly conserved ATP-dependent proteases and its components (*Lonp1, Clpxp, Yme1l1, Afg3l2,* and *Spg7*) are robustly induced in aged hearts [Figure 2A]. This finding implies the development of stress in the heart during aging, and these proteases play a significant role in maintaining mt proteostasis, indicating a heightened response to aging-associated accumulation of abnormal or impaired protein function in mitochondria. Importantly, *LONP1* and *CLPP* play essential roles in maintaining mitochondrial function and integrity in the matrix, particularly under stress conditions^[22,23]^. We have observed that their upregulation in aged hearts could reflect a response mechanism to the increased mitochondrial stress within the matrix. This finding is supported by a recent study that shows cooperative regulation of mitochondrial proteostasis by *Lonp1* and *Clpp*, particularly under conditions that induce proteotoxic stress^[24]^. Furthermore, disruption of *Lonp1* impairs normal development, fecundity, and lifespan, demonstrating the crucial role of LONP1 protein in maintaining mitochondrial function and organismal health^[25,26]^. The location of these proteases in the mitochondria states the importance of their respective quality control areas. Additionally, *Lonp1* deletion induces mitochondrial unfolded protein response (UPR^mt^) and activates antioxidant and stress response mechanisms, which could have parallels in human cardiac aging^[25]^. We have also recently shown that *Lonp1* plays an essential role in myocardial stress response and is protective against ischemia-reperfusion-induced oxidative injury^[27]^. Similarly, the upregulation of mitochondrial inner membrane ATP-dependent protease components like *Afg3l2, Yme1l1, and Spg7* in the aged heart aligns with the known roles of these proteases in the quality control of mitochondrial inner membrane proteins^[28,29]^. Although *Afg3l2* has a *Chop* element in its promoter, the mechanisms behind *Yme1l1* and *Spg7* induction need investigation^[30]^. These findings imply a robust response to aging-associated stress, indicating an adaptive mechanism to cope with potential damage or alterations in imported mitochondrial proteins, providing a novel area of therapeutic strategies in preserving mitochondrial function during aging^[31]^.

In contrast to mitochondrial proteases, mitochondrial chaperones (*Hspa9/Grp75, Hspd1/mtHsp60*) did not show significant changes between aged and younger hearts but showed significant induction of *Dnaja2*, a mitochondrial co-chaperone, indicating prominent mitochondrial stress in the heart during aging. This insight implies that the other chaperones may already be equipped to handle stress during aging. Previous studies have shown that mitochondrial chaperones such as *Hspa9* protect against oxidative stress-induced mitochondrial dysfunction, and *Hspd1* is crucial for protein folding^[32,33]^. This suggests a distinct regulatory mechanism for chaperones, which is required to properly fold and assemble newly imported proteins in the mitochondria compared to proteases during aging. Additionally, we observed a substantial increase in *Atf5* in 25-month hearts compared to 10-month hearts. This is in line with the role of *Atf5* in mitochondrial stress response, as detailed in studies by Fiorese *et al.* (2016), which emphasized ATF5’s importance in mitochondrial function and stress response^[34]^. *Atf-5* is a significant regulator of mitochondrial unfolded protein response (UPR^mt^), promoting the induction of mitochondrial proteases and chaperones to restore mitochondrial proteostasis^[34,35]^. One hypothesis is that chaperones in the heart maintain a sufficient baseline capacity to handle stress without further upregulation, as seen in non-replicating cells like neurons^[36]^. The stability of chaperone levels might reflect an adaptive response to prevent mitochondrial dysfunction, supported by studies showing enhanced chaperone expression protects against cardiac stress^[37]^.

Mitochondrial dynamics involve fission and fusion to ensure that each mitochondrion receives certain copies of the genome and other contents, where DNML1 protein plays an indispensable role in maintaining mitochondrial integrity through fission along with the coordination of *Mfn1* and *Fis1* expression^[38]^. However, a notable increase in *Dnm1l* expression in aged hearts supports the hypothesis that enhanced mitochondrial fission, regulated by *Dnm1l*, may serve as a compensatory mechanism in aging cells for maintaining mitochondrial quality^[39,40]^. Another study suggests a decline in the expression of fission-related proteins, including *Dnm1l*, in the aging heart, which could lead to an imbalance in mitochondrial dynamics favoring fusion over fission^[41,42]^. However, the downregulation of *Mfn1* and *Fis1* observed in the aging hearts, along with substantial induction of Mfn2, implies an impaired overall fusion and fission process, as an increase in *Dnm1l* expression may be to compensate for the impaired fusion and fission process. An induction of *Pink1*, a mitochondrial-specific autophagy mediator, without the induction of *Atg7*, a global autophagy mediator, was noted, suggesting that mitochondrial dynamics are more prone to age-associated changes than global organelle recycling. Our findings on mitochondrial dynamics reveal a notable dysregulation in genes related to fission and fusion during cardiac aging. The observed increase in *Dnm1l* expression, coupled with the downregulation of *Mfn1* and *Fis1*, suggests structural remodeling of mitochondria in aged hearts. This imbalance may indicate a compensatory mechanism to maintain mitochondrial quality through enhanced fission, potentially at the expense of impaired fusion processes, highlighting the structural adaptations that occur in response to aging-associated stress.

The ER, like mitochondria, is a crucial organelle involved in synthesizing and folding proteins, but its susceptibility to dysfunction with age and the accumulation of misfolded proteins, leading to ER stress, is less understood. The coordination between mitochondrial and ER functions in cellular processes is well-established^[17,43]^. Aging activates the *Perk* (*Eif2ak3*)*, Atf6,* and *Ire1α-Xbp1* stress pathways, suggesting adaptive mechanisms to restore ER proteostasis. Specifically, our findings show significant upregulation of *Xbp1* transcripts, a key transcription factor regulating ER stress response genes, in aged hearts compared to young hearts [Figure 3A]^[44]^. This suggests an activated unfolded protein response (UPR) to restore ER homeostasis under stress^[15,17]^. Consistent with Baek *et al*. (2012), our results emphasize *Xbp1*’s critical role in addressing age-related ER stress^[45]^. However, in contrast to Lee *et al*. (2015), who reported diminished *Xbp1* efficacy with age, our findings suggest its upregulation persists in aged hearts^[46]^. Interestingly, *Atf6* activation was observed alongside *Xbp1* induction but not with *Perk*, which is critical for cardiomyocyte survival under stress^[47,48]^. The absence of PERK signaling may impair protein synthesis regulation, increasing vulnerability to acute stressors^[49]^. We also investigated the mRNA levels of heat shock molecular chaperones *Hspa5, Dnajb9*, and *Hsp90b1*. Our analysis revealed a significant reduction in *Hsp90b1* induction, which is essential for protein folding. This suggests a compromised stress-handling capacity, aligning with studies showing its overexpression is cardioprotective^[50]^. Meanwhile, *Hspa5* and *Dnajb9* transcript levels remained unchanged, indicating selective regulation of chaperones in the aging heart’s ER stress response. Although *Atf6* mRNA showed an increase, it was not statistically significant, and no changes were observed in *Atf3* or *Atf4* mRNAs, implying their limited role in cardiac aging. PERK-mediated global translation inhibition during ER stress may also favor *Atf-5* translation, enhancing specific ER and mitochondrial stress genes^[51,52]^. These findings highlight a mixed ER stress response in aging hearts, with some components upregulated and others downregulated, necessitating further research into enhancing ER stress response capacity.

The observed decline in *Mtor and Nrf1* induction in aged hearts aligns with existing literature, emphasizing these pathways’ significance in maintaining cardiac function and metabolic homeostasis. *Mtor*, a central regulator of cell growth and protein synthesis, has been shown to decrease with age, contributing to impaired autophagy and increased susceptibility to stress^[53]^. Similarly, *Nrf1*, a critical player in oxidative stress response and mitochondrial biogenesis, is crucial for maintaining cellular homeostasis, and its reduction has been linked to diminished mitochondrial function and increased oxidative damage in aging cardiac tissue^[54]^. The unchanged *AMPK1* (*Prkaa1*) levels suggest that while energy balance mechanisms might remain intact, the decline in *Mtor* and *Nrf1* likely disrupts other crucial metabolic and stress response pathways. These findings are consistent with the concept that aging is associated with accumulating damaged proteins and organelles and contributing to age-related cardiac dysfunction^[55]^. A marked increase in oxidative damage markers, such as 4-hydroxynonenal (4-HNE), underscores the heightened mt and ER stress in aging cardiac tissue. However, the increased mt and ER stress response, specifically through mt ATP-dependent proteases and *Xbp1*, needs deeper investigation to identify how aging activates these stress responses and whether they are beneficial or detrimental. This finding is consistent with our observation of increased oxidative damage in older hearts, as evident from the 4HNE protein adduct levels [Figure 5]. This age-associated oxidative stress marker is a critical factor in cardiac aging that predispose to various pathological abnormalities that align well with the findings^[56]^.

**Figure 6.**
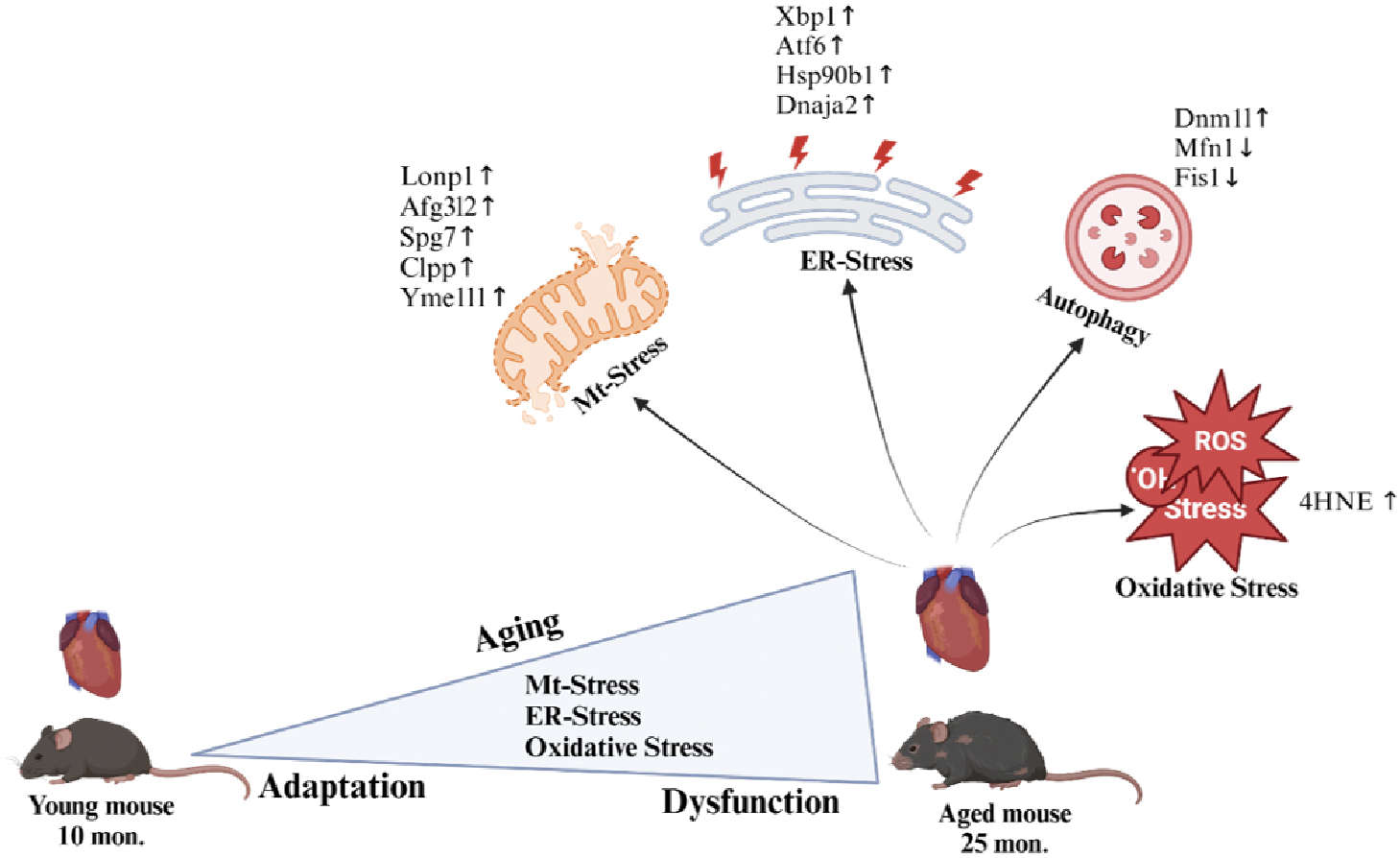
Aging induces mt and ER stress responses in the heart. During cardiac aging, mitochondria (mt) and the endoplasmic reticulum (ER) are two important organelles exposed to high energy demand. Initially, they adapt by upregulating the stress response, but as aging progresses, oxidative stress and damage increase, thereby predisposing the heart to dysfunction. Created using BioRender (www.app.biorender.com).

In conclusion, our study provides critical insights into the stress response in cardiac aging, focusing on the interplay between mt and ER stress responses [Figure 6]. Although no significant role of mitochondrial biogenesis was involved, decreased fission and fusion processes during aging to maintain ordinary mitochondrial division warrants further investigation. Notably, a group of mitochondrial ATP-dependent proteases such as *Lonp1, Yme1l1, Clpxp, Afg3l2,* and *Spg7* were upregulated in aged hearts, indicating a heightened stress response that this organ develops during aging. Whether this leads to ER stress load through a feedback regulation requires investigation. A significant increase in *Xbp1* induction in ER stress shows an interconnected regulation with the mitochondrial stress response. Our findings on the interplay between mitochondrial (mt) and endoplasmic reticulum (ER) stress responses in cardiac aging have significant translational implications. The observed upregulation of mitochondrial proteases such as Lonp1 and ER stress mediator *Xbp1* highlights potential therapeutic targets for mitigating aging-associated cardiac dysfunction. Enhancing *Lonp1* expression or activity could bolster mitochondrial proteostasis, reducing oxidative stress and promoting the clearance of damaged proteins, while pharmacological activation of *Xbp1* might alleviate ER stress and restore proteostasis in the aging myocardium. These interventions could prevent adverse cardiac remodeling and preserve cardiac function in aging hearts. Furthermore, the identification of these adaptive stress responses paves the way for developing precision therapies that integrate molecular findings with clinical data, aiming to delay or prevent the progression of age-related heart failure. Understanding these hallmark pathways ultimately provides a critical foundation for designing novel strategies to address mitochondrial and ER dysfunction in aging populations, bridging the gap between basic research and clinical application. Overall, the current study enhances our understanding of cardiac aging by identifying critical stress response pathways activated in aged hearts and setting a foundation for future exploration into the mechanisms and potential therapeutic targets for age-related cardiac dysfunction.

### Limitations and future directions

Our study on cardiac aging provides valuable insights but has certain limitations. These include the specificity of the model, which may affect the broader applicability of the findings. A key limitation is that we focused only on gene expression at the transcript level, which, while providing the advantage of identifying early and dynamic molecular changes, does not capture post-transcriptional modifications or protein-level alterations that may further influence cellular function. Despite this, emphasizing transcript measurements enables us to uncover upstream regulatory pathways and early markers of aging-related mitochondrial and ER stress, offering a foundation for future protein-level investigations. Future research will address these limitations by applying findings to broader models, including human tissues, and conducting longitudinal and functional studies to better understand the progression and implications of molecular changes in cardiac aging. Investigating selective gene expression and exploring therapeutic interventions targeting identified pathways could yield significant advances. Additionally, integrating these molecular findings on the interplay of mitochondrial and endoplasmic reticulum stress pathways in cardiac aging with clinical data will enhance the practical application of this research in healthcare, particularly for the aged population. Addressing these aspects will deepen our understanding of cardiac aging and aid in developing effective treatments for age-related cardiac dysfunction.

Building on our findings, follow-up studies could explore pharmacological activators or gene therapy approaches to enhance LONP1 expression/activity or stimulate *Xbp1* signaling in preclinical models of cardiac aging. Additionally, testing combinatorial therapies that simultaneously target both mt and ER stress pathways may provide synergistic benefits in mitigating oxidative damage, restoring proteostasis, and preserving cardiac function. Longitudinal studies integrating molecular, functional, and imaging approaches could also help establish the efficacy and safety of these interventions over the aging continuum. Finally, translating these insights to human cardiac tissues or organoid models could provide a vital step toward clinical applications, helping to bridge the gap between basic science and therapeutic strategies for aging-associated cardiac dysfunction.

## Supplementary Material

Supplementary

## Figures and Tables

**Table 1.** List of TaqMan gene expression assays employed in the study

Gene	Assay ID

*mt-Cytb*	Mm04225271_g1
*mt-Co2*	Mm03294838_g1
*Dnm1l*	Mm01342903_m1
*Afg3l2*	Mm01258204_m1
*Spg7*	Mm00462653_m1
*Clpp*	Mm00489940_m1
*Lonp1*	Mm01236887_m1
*mt-Co1*	Mm04225243_g1
*Tfam*	Mm00447485_m1
*Ndufs4*	Mm00656176_m1
*mt-Nd4*	Mm0060082_m1
*mt-Atp6*	Mm0060077_m1
*Clpx*	Mm00488586_m1
*Atf3*	Mm00476033_m1
*Atf4*	Mm00515325_m1
*Atf5*	Mm00459515_m1
*Atf6*	Mm0053948_m1
*Xbp1*	Mm00457357_m1
*Ddit3*	Mm01135937_m1
*Hspa9*	Mm00477716_g1
*HSpd1*	Mm00849835_g1
*Tid1*	Mm07297914_m1
*Hspa5*	Mm00517691_m1
*Hsp90b1*	Mm00441926_m1
*Dnaja2*	Mm00444898_m1
*Dnajb9*	Mm01622956_s1

## Data Availability

The raw data supporting this study’s findings are available within this Article and its Supplementary Material. Further data are available from the corresponding author upon reasonable request.
