# Supplementary figures and images for: Aging triggers mitochondrial, endoplasmic reticulum, and metabolic stress responses in the heart
Source: J Cardiovasc Aging. Author manuscript; Available in PMC 2025 Mar 21. (PMC11928159; doi:10.20517/jca.2024.17)

Supplementary Figure 1

07/12/21

10ug loading

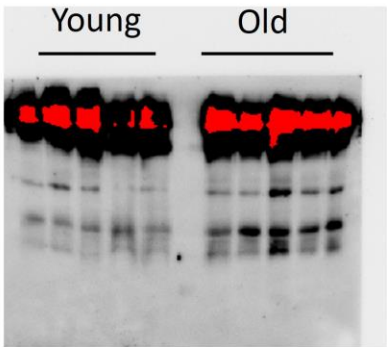

4-HNE- L.M wt (20-75 Kda)

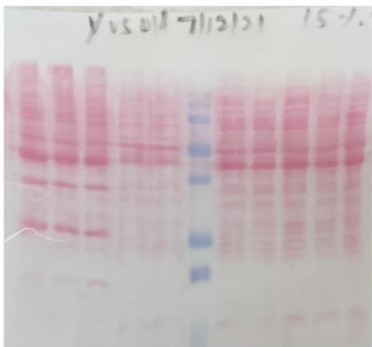

Ponceau stain

Supplement: Supplementary [file NIHMS2059148-supplement-Supplementary.zip › jca4017-SupplementaryMaterials.pdf]
